# Hormonal Status of Transgenic Birch with a Pine Glutamine Synthetase Gene during Rooting In Vitro and Budburst Outdoors

**DOI:** 10.3390/biom13121734

**Published:** 2023-12-02

**Authors:** Vadim G. Lebedev, Alla V. Korobova, Galina V. Shendel, Konstantin A. Shestibratov

**Affiliations:** 1 Branch of the Shemyakin-Ovchinnikov Institute of Bioorganic Chemistry of the Russian Academy of Sciences, 142290 Pushchino, Russia; schestibratov.k@yandex.ru; 2 Ufa Institute of Biology of the Ufa Federal Research Center of the Russian Academy of Sciences, 450054 Ufa, Russia; muksin@mail.ru (A.V.K.); fedorov@anrb.ru (G.V.S.)

**Keywords:** abscisic acid, auxin, *Betula*, budburst, glutamine synthetase, N deficiency, phosphinothricin, rooting in vitro, transgenic trees

## Abstract

Improving nitrogen use efficiency (NUE) is one of the main ways of increasing plant productivity through genetic engineering. The modification of nitrogen (N) metabolism can affect the hormonal content, but in transgenic plants, this aspect has not been sufficiently studied. Transgenic birch (*Betula pubescens*) plants with the pine glutamine synthetase gene *GS1* were evaluated for hormone levels during rooting in vitro and budburst under outdoor conditions. In the shoots of the transgenic lines, the content of indoleacetic acid (IAA) was 1.5–3 times higher than in the wild type. The addition of phosphinothricin (PPT), a glutamine synthetase (GS) inhibitor, to the medium reduced the IAA content in transgenic plants, but it did not change in the control. In the roots of birch plants, PPT had the opposite effect. PPT decreased the content of free amino acids in the leaves of nontransgenic birch, but their content increased in GS-overexpressing plants. A three-year pot experiment with different N availability showed that the productivity of the transgenic birch line was significantly higher than in the control under N deficiency, but not excess, conditions. Nitrogen availability did not affect budburst in the spring of the fourth year; however, bud breaking in transgenic plants was delayed compared to the control. The IAA and abscisic acid (ABA) contents in the buds of birch plants at dormancy and budburst depended both on N availability and the transgenic status. These results enable a better understanding of the interaction between phytohormones and nutrients in woody plants.

## 1. Introduction

Increasing crop productivity is one of the main research directions of plant genetic engineering. It is achieved by enhancing photosynthesis, manipulating phytohormone content, or improving plant nutrition [1,2,3]. In the latter approach, research has mainly focused on increasing the nitrogen use efficiency (NUE), as this element is a key nutrient for plant growth and development. This has been implemented through the transfer of various structural and regulatory genes associated with the uptake, transport, and assimilation of N in plants [4,5]. Among these, special attention has been paid to glutamine synthetase (GS; EC 6.3.1.2) genes due to the important roles of this enzyme in N assimilation in plants [6].

Nitrogen is part of compounds such as amino acids, nucleic acids, chlorophyll, and phytohormones. These substances make up only a small fraction of plant mass, but they are extremely physiologically important [7]. Amino acids are a substrate for the synthesis of structural proteins and enzymes catalyzing biochemical processes, chlorophyll plays a key role in photosynthesis, and phytohormones regulate the growth and development of plants. Therefore, a modification of N metabolism can, besides accelerating growth, affect other vital processes. Transgenic plants with modified N metabolisms have been demonstrated to have increased resistance to various abiotic stresses [8,9,10], accelerated flowering [11,12], decreased anthocyanin content [13], and altered secondary cell wall formation [14]. The modification of the N metabolism can also affect the hormonal statuses of plants: transgenic plants show changes in the contents of auxins [14], cytokinins [15], and abscisic acid (ABA) [16]. Such studies are scarce, however, and the plants’ hormonal contents have been investigated separately from the directly related processes, e.g., the contents of auxins and rooting.

Valuable genotypes of trees, including transgenic ones, should preferably be vegetatively propagated to preserve their important properties. Among vegetative propagation methods, which include budding, grafting, layering and cutting, the most effective and economical technique for woody plants is propagation via stem cutting. For example, the propagation of elite genotypes of eucalyptus via cutting is the main stage in the creation of high-yielding forest plantations [17]. A central role in cutting propagation is played by adventitious rooting, and a key factor in this process is auxins [18]. The leaves of one of the two transgenic lines of hybrid poplar with the *GS1* gene have been found to experience a significant increase in the level of indoleacetic acid (IAA) [19]; however, no rooting studies have been carried out.

In boreal and temperate trees, winter dormancy is an important adaptive mechanism of survival under unfavorable conditions. This complex process depends on many internal and external factors, and our knowledge about the dormancy release and budburst is very limited in perennial plants [20]. Plant hormones play critical roles in it; in particular, ABA participates in the establishment, maintenance and release of bud dormancy [21]. ABA is also a main regulator of drought stress [22,23,24]. There is a strong interaction between phytohormones and nutrients, but its mechanisms are still not completely clear [25]. ABA is possibly involved in increasing drought tolerance in herbaceous [10] and woody [26] plants with improved NUE; however, no studies of this subject have been conducted.

Birch species (*Betula* L.) are widespread in northern temperate and boreal forests [27]. In these regions, birch can be used as a plantation alternative to the more thermophilic eucalyptus. To increase its productivity, the gene of a cytosolic form of GS from the Scots pine has previously been transferred to the downy birch [28]. These plants demonstrated enhanced growth [29], earlier flowering [5], and changes in N and K contents in soil after a 4-year pot experiment [30]. The purpose of the present study was to investigate the hormonal compositions of transgenic birch plants as follows: (1) In the process of rooting in vitro, including exposure to phosphinothricin (PPT), a GS inhibitor and an active ingredient of nonselective, broad-spectrum herbicides [31]. PPT was included in this study to determine the relationship between possible changes in hormone levels and increased GS synthesis in transgenic plants. (2) Moreover, we studied these hormonal compositions during the dormancy release after three years of outdoor cultivation under different N availability conditions.

## 2. Materials and Methods

### 2.1. Plant Material

Transgenic birch (*Betula pubescens* Ehrh.) plants were obtained via the *Agrobacterium*-mediated transformation of leaf explants of the Bp3f1 genotype with the pGS vector [28]. The pGS vector contains the nos-*nptII* gene and *GS1* gene, encoding the cytosolic form of GS from *Pinus sylvestris* under the CaMV 35S promoter. Three transgenic lines, containing the *GS1* gene—F14GS8b, F14GS9b and F14GS2b (also referred as GS 8b, GS 9b, and GS 2b)—were selected for in vitro experiments. These lines were characterized by accelerated or similar growth, and a dwarf phenotype relative to the wild type, respectively [30]. The plants were micropropagated on WPM medium supplemented with 0.3 mg/L benzyladenine, which was excluded from the culture medium at the last passage before rooting. After proliferation, the microshoots were planted in rooting medium (hormone-free WPM medium).

### 2.2. In Vitro Experiments

In a preliminary experiment to evaluate the inhibitory concentration of PPT, wild-type plants were planted on a medium containing 0, 0.01, 0.02, 0.05, 0.1, 0.2, and 0.05 mg/L PPT. Each treatment included 3 replicates of 18 shoots. In the main experiment, transgenic and control birch plants were rooted on a medium with 0 or 0.1 mg/L PPT (the minimum concentration that causes inhibition of root formation in nontransgenic birch plants). Each treatment included 4 replicates of 18 shoots. All media contained 30 g/L sucrose and 7 g/L agar. The plants were cultivated at 22–24 °C and a 16 h light/8 h dark photoperiod with a light intensity of 23–27 μmol m^−2^ s^−1^. The rooting frequency and number of roots per shoot were determined during 2 weeks of cultivation. Leaf and root samples were frozen in liquid nitrogen, lyophilized, and used for further analyses.

### 2.3. Pot Experiment

Micropropagated birch plants (wild type and transgenic line GS 8b) after adaptation to the greenhouse conditions were transplanted into pots with peat:perlite (3:1) and transferred outdoors. The plants were grown for three years and overwintered under natural conditions. The average air temperature during the growing season (May–September) varied from 15.8 to 16.8 °C, and the amount of precipitation varied from 277 to 434 mm. During the summer, the plants were fertilized weekly with solutions of macro- and micronutrients containing 0 or 10 mM N in calcium nitrate form. At the end of the 3rd growing season, the plant height and stem base diameter were measured. Budburst was assessed every 2–3 days during the spring of the 4th year. Bud break was defined as a stage when new green foliage was clearly visible from the bud scales. Each treatment included from 4 to 7 replicates (trees) of 12 lateral buds. Lateral buds at the dormancy stage and stage 3 (the appearance of green foliage) according to Murray et al. [32] were frozen in liquid nitrogen, lyophilized, and used for further analyses.

### 2.4. Amino Acid Analysis

The amino acid contents in birch microshoots were determined via ion exchange chromatography with a Biochrom 30 amino acid analyzer (Biochrom Ltd., Cambridge, UK) using a system of lithium citrate buffers as described previously [33].

### 2.5. Hormone Extraction and Purification

Extraction of hormones from lyophilized explants and buds was carried out using 70% ethanol overnight at 4 °C. After centrifugation and alcohol evaporation, the aqueous residue was brought to 9 mL with distilled water and acidified with hydrochloric acid to pH 2–3. Hormone purification was performed as described [34]. ABA and IAA were extracted twice with diethyl ether (3 mL each) and then transferred to sodium bicarbonate (3 mL). After acidifying the soda, the hormones were extracted again with ether (twice 1.5 mL each) and methylated with diazomethane. At each stage, the hormones concentrated due to a decrease in the amount of extractant, which increased the selectivity of hormone recovery [35].

### 2.6. Immunoassay

The dry residue after the evaporation of ether was dissolved in 100 μL of 80% ethanol, and 10 μL was added to the wells of microplates after preliminary adsorption on the walls of the conjugated corresponding hormone with protein. ABA and IAA standards in a series of tenfold dilutions were added to a portion of the wells to subsequently obtain a calibration curve. Specific serum was added to each well and incubated. Unbound rabbit antibodies were washed away, and goat anti-rabbit IgG, conjugated to peroxidase, was incubated with the adsorbed antigen–antibody complex. Finally, a substrate solution consisting of o-phenylene-diamine was added. The color developed was quantitated at 492 nm with a microphotometer. A more detailed protocol can be found in the methodological article [35].

### 2.7. Statistical Analysis

Data are presented as means ± standard errors (SE). All data were tested via ANOVA using the Statistica 10 software (StatSoft, Tulsa, OK, USA). The means were separated using Duncan’s multiple range test at a significant level of 0.05. A paired *t*-test was used to test for differences in the growth parameters between the control and transgenic birch lines.

## 3. Results

### 3.1. Effect of PPT on Rooting Birch In Vitro

**Figure 1:**
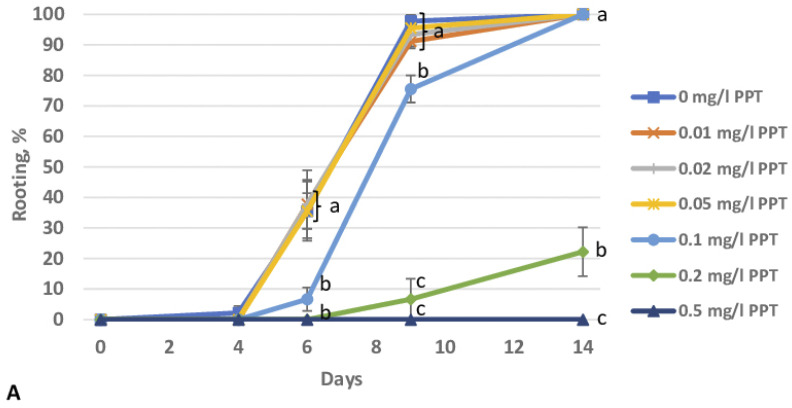

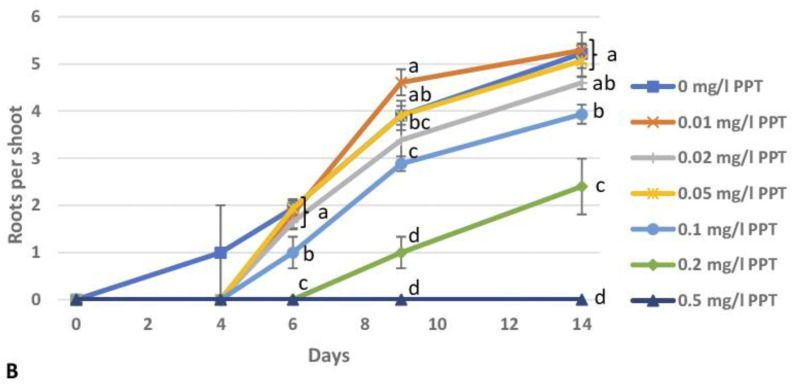
Effect of phosphinothricin (PPT) on root formation of wild-type birch microshoots. (**A**) Rooting frequency; (**B**) number of roots per shoot. Data are means ± SE of three replicates (18 shoots per replicate). Different letters indicate significant differences between treatments (*p* < 0.05).

To assess the effect of PPT on root formation in birch plants in vitro, we determined a rooting-inhibiting concentration in a preliminary experiment. The results show that in an experiment with nontransgenic plants, 0.01–0.05 mg/L PPT did not affect the rooting in vitro (Figure 1A). On a medium with 0.1 mg/L PPT, the rooting was substantially slowed down at the early stages, but after two weeks, all shoots were rooted. A concentration of 0.2 mg/L PPT significantly slowed down the rooting (only 22.2% of shoots were rooted after two weeks), and no rooting took place at 0.5 mg/L PPT. The concentration of PPT had a similar effect on the number of roots per shoot (Figure 1B). For further experiments, we chose a concentration of 0.1 mg/L PPT, the minimum concentration with an inhibitory effect on root formation in nontransgenic birch plants.

**Figure 2:**
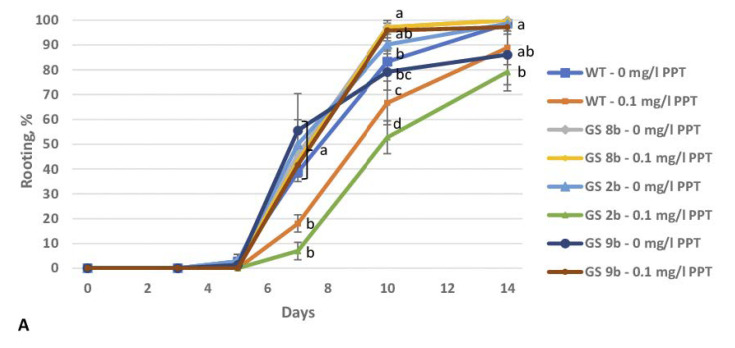

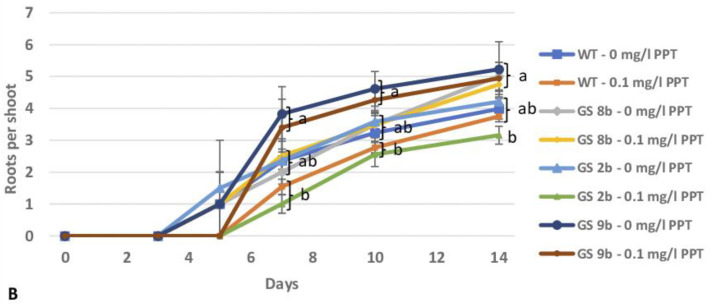
Root formation of birch microshoots at 0 and 0.1 mg/L PPT. (**A**) Rooting frequency; (**B**) number of roots per shoot. Data are means ± SE of four replicates (18 shoots per replicate). Different letters indicate significant differences between treatments (*p* < 0.05).

**Figure 3:**
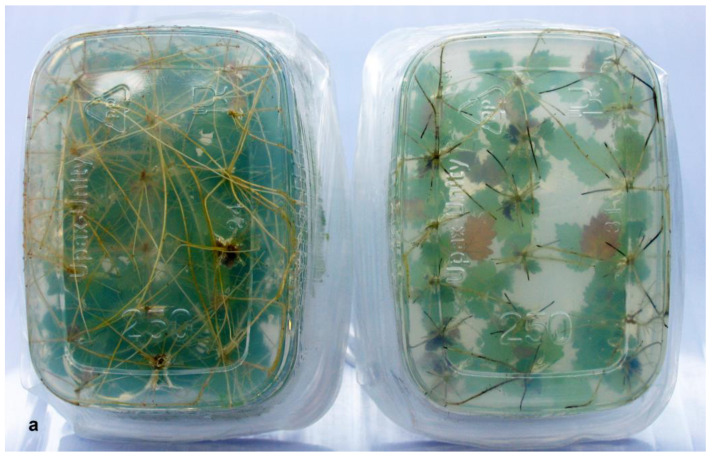

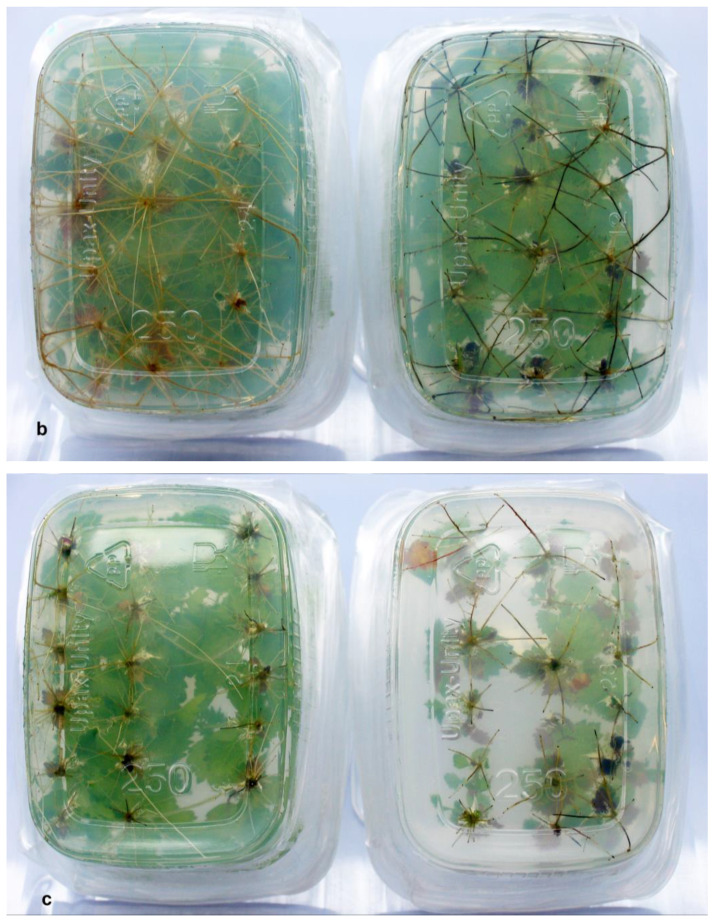
Rooting of birch on a medium without PPT (left) and with 0.1 mg/L PPT (right). (**a**) Wild type; (**b**) line GS 8b; (**c**) line GS 2b.

Three birch lines with the *GS1* gene were used to assess the effect of PPT on the rooting of transgenic plants. On a medium without PPT, these lines did not differ significantly from the control in terms of rooting frequency (Figure 2A) and the number of roots per shoot (Figure 2B). However, the roots of line GS 2b were significantly shorter as compared to the control and other transgenic lines (Figure 3). On a medium with 0.1 mg/L PPT, the rooting of nontransgenic plants was significantly lower as compared to the control, but the difference was significant only seven days after planting (Figure 2A). Unlike the wild type, the rooting of line GS 2b was significantly lower throughout the entire experiment. The addition of PPT to the medium did not change the rooting frequency of transgenic lines GS 8b and GS 9b. PPT had almost no effect on the number of roots formed in birch microshoots: only line GS 2b featured their considerable decrease, significant seven days after the rooting (Figure 2B). In addition, considerably shorter dark-colored roots formed on a medium with PPT in all genotypes (Figure 3). Two-factor analysis showed that after seven and ten days, the rooting frequency was significantly influenced by the genotype, the PPT, and their interaction; the genotype alone had a significant effect on the number of roots per shoot.

### 3.2. IAA and Amino Acid Content in Tissues of Birch Plants In Vitro

**Figure 4:**
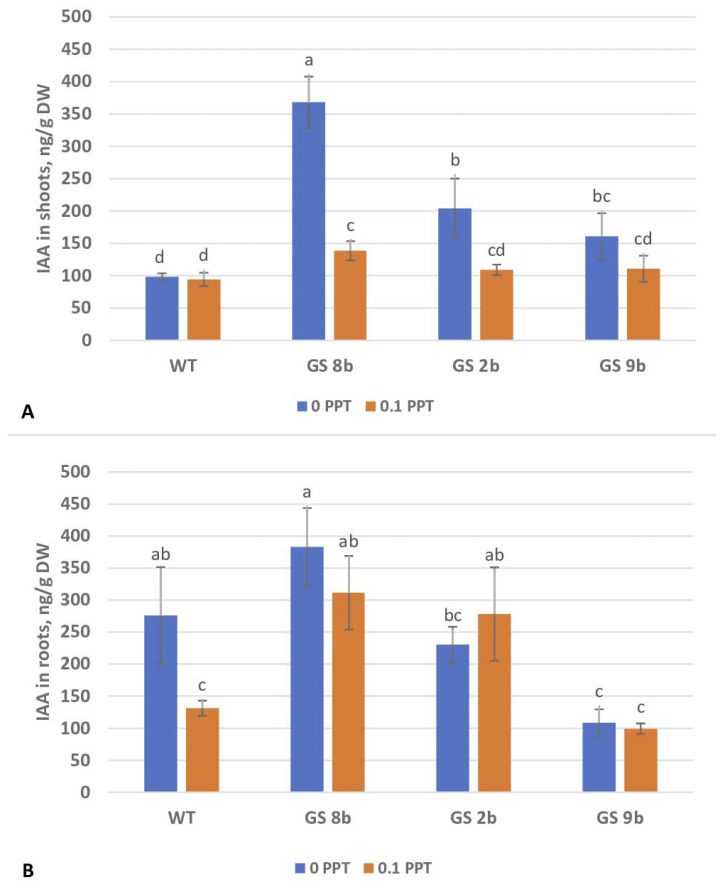
Concentration of indoleacetic acid (IAA) in birch plants on the 10th day of rooting. (**A**) Shoots; (**B**) roots. Different letters indicate statistically significant differences at *p* < 0.05.

For the rooting without PPT, the content of IAA in shoots of all transgenic lines was significantly higher than in the control (about three times higher in line GS 8b) (Figure 4A). The addition of PPT to the medium did not change the content of IAA in the microshoots of the control, but significantly reduced it in the transgenic lines. Despite this decrease, the content of IAA in GS 8b plants on a medium with PPT was still significantly higher than in the control plants (by 46.8%). The addition of PPT reduced the IAA content in the roots of nontransgenic birch by half, but in the roots of transgenic plants, it did not change significantly (Figure 4B).

The assessment of the content of amino acids in birch plants in vitro showed that their total content in transgenic line GS 8b barely changed as compared with the control (was 3% higher) without PPT in the medium, but the contents of glutamine, aspartic acid, and glutamic acid increased significantly (by 46.6%, 29.5%, and 12.1%, respectively) (Table 1). The addition of PPT reduced the amino acid content in the control birch by 16.7%, but in the transgenic line, it increased by 15.4%.

### 3.3. Effect of N Availability on Growth and Budburst of Birch Plants

Plants of the wild type and of transgenic line GS 8b were grown outdoors for three years under different N availability conditions: the fertilizing solution contained 0 or 10 mM of N. The transgenic plants significantly exceeded the control by their height, diameter, and stem volume in the absence of N in the nutrition solution, but did not differ when grown with a sufficient N supply (Table 2).

**Figure 5:**
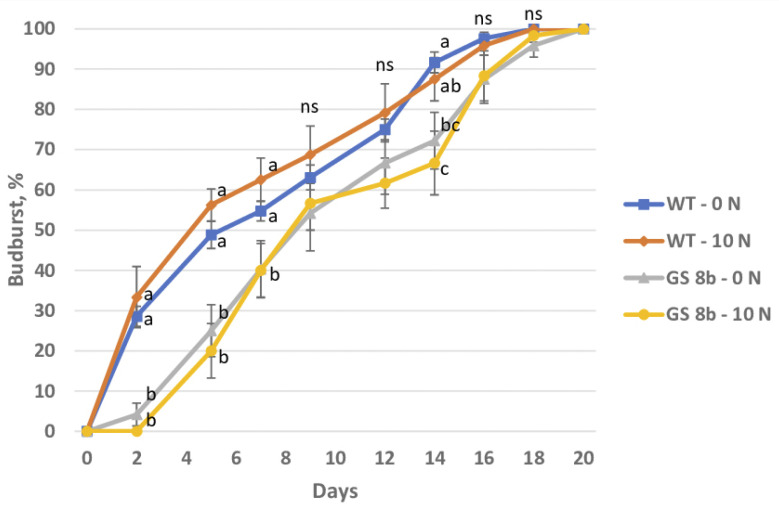
Effect of N supply on budburst of birch plants. Data are means ± SE of 4–7 biological replicates (12 buds per replicate). Different letters indicate significant differences between treatments (*p* < 0.05); ns, not significant.

In the spring of the fourth year of growth, we evaluated the budburst rate of the control and transgenic birch plants. Nitrogen availability did not affect the budburst; however, we observed a delay in the budburst in the transgenic plants as compared to the control (Figure 5). The transgenic trees budded three days later on average.

### 3.4. IAA and ABA Content in Buds of Birch Plants

**Figure 6:**
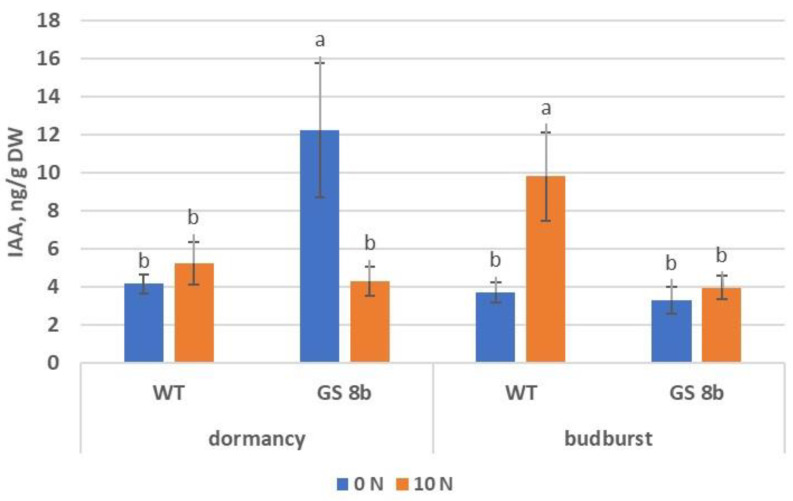
Contents of IAA in buds of birch plants. Different letters indicate statistically significant differences at *p* < 0.05.

Buds of birch plants were evaluated for the contents of IAA and ABA in two phases of development: dormancy and appearance of green foliage. In dormancy, the contents of IAA were similar in the control (at two N concentrations) and in line GS 8b (at 10 mM of N), but IAA in the transgenic line without N was significantly (2.9-fold) higher (Figure 6). In the process of budburst, the content of IAA did not change significantly in the control at zero N and in line GS 8b at 10 mM N. At the same time, the control featured a sharply increased IAA content under sufficient N conditions, and in the transgenic line, we found a sharply decreased IAA under N deficiency.

**Figure 7:**
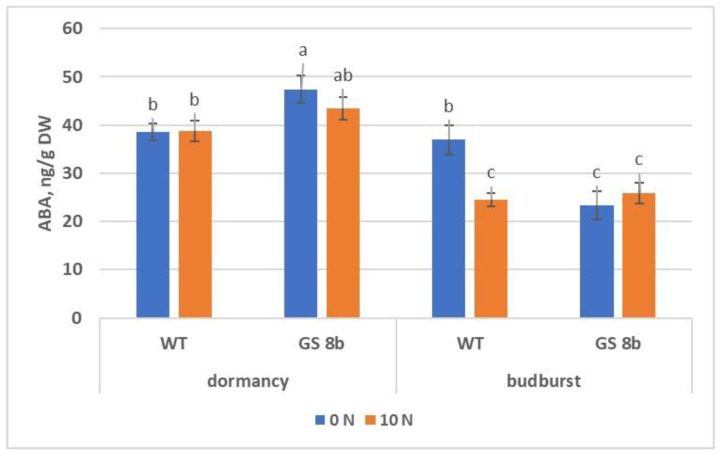
Content of abscisic acid (ABA) in buds of birch plants. Different letters indicate statistically significant differences at *p* < 0.05.

In the state of dormancy, the ABA content in the buds of the control did not depend on N availability during the growth, and it was slightly lower than in the transgenic plants against a sufficient N background and significantly lower compared to plants with N deficiency (Figure 7). During the budburst, the ABA content significantly decreased in the transgenic plants at both N concentrations and in the control at a sufficient N, whereas with a lack of N, it did not change in the control.

## 4. Discussion

### 4.1. Auxin Status of Birch Plants during Rooting In Vitro

Auxin plays a key role in the growth and development of plants, participating in processes such as organogenesis, differentiation of vascular tissues, apical dominance, initiation of root development, and tropisms, as well as cell extension, division, and differentiation [36,37,38]. The content of auxin is related to the N status of plants since amino acid tryptophan is a key metabolite and precursor of auxin [39]. The main enzyme of N metabolism in plants is GS, which catalyzes the synthesis of glutamine from glutamate and ammonia [40], and the genes encoding it are often used in genetic engineering for improving the NUE. *L*-PPT, an amino acid from the soil bacteria *Streptomyces* spp., is a structural analog of glutamate and irreversibly inhibits GS, leading to plant death [41]. We evaluated the auxin content in transgenic birch plants with the pine cytosolic *GS1* gene during rooting and under the influence of PPT.

In a preliminary experiment, we determined that the inhibition of rooting of nontransgenic birch microshoots occurred at 0.1 mg/L PPT (Figure 1A). The insertion of the *GS1* gene did not alter the rooting of birch plants as compared to the control. However, the addition of a GS inhibitor to the medium led to a noticeable decrease in the rooting in nontransgenic plants and line GS 2b, whereas in transgenic lines GS 8b and GS 9b, it did not change (Figure 2A). PPT had a small influence on the number of formed roots. The effect of PPT also manifested itself in a decrease in the length of the roots and a change in their color (Figure 3).

The content of IAA was significantly (1.5- to 3-fold) higher in shoots of all transgenic birch lines as compared to the control (Figure 4). This increase is greater than that previously reported for transgenic poplar and tobacco plants with the *GS* genes, by 3.8–32.2 and 15.6%, respectively [14,19]. In these transgenic plants, the expression of the gene *ASA1*, which catalyzes the IAA biosynthesis stage, increased. In addition, the contents of free Glu and free Gln significantly increased in transgenic poplar and tobacco plants [14,19], which is in good agreement with our data. However, the total amino acid content in birch increased by only 3%, while in poplar, it increased by 19.5%, and in tobacco, by 43.6%. Due to the fact that the initial stage of IAA biosynthesis is the production of anthranilate from glutamine and chorismate [19], the increased concentration of IAA in the tissues of the transgenic plants can be caused exactly by an increase in the glutamine content. The increased content of glutamic acid in the transgenic plants also indicates its possible role in the regulation of auxin synthesis. The reduced sensitivity to glutamate found in the auxin transport mutant is indicative of a link between the signaling of glutamate and auxin [42]. It should be mentioned that transgenic plants with the *GS* gene have an increased content of those amino acids that play an important role in the transport and remobilization of N in plants [43].

The addition of PPT decreased the accumulation of IAA in shoots of all transgenic lines down to the level of nontransformed plants, which confirmed the participation of GS in increasing the level of auxins. Thus, an increase in the rooting rate of transgenic birch lines GS 8b and GS 9b on a medium without PPT can be a consequence of an increased content of auxins in them. At the same time, other factors could be involved in the rooting, since lines GS 8b and GS 9b rooted better on a medium with PPT than the control, but only line GS 8b had a significantly higher content of IAA than the control. For this reason, line GS 8b was chosen for further in vivo studies. The decrease in rooting in line GS 2b was most likely associated with serious phenotype disorders (dwarfism, etc. [30]), but not with IAA, as this line did not differ in its content from lines GS 8b and GS 9b with significantly better rooting ability. PPT reduced the IAA content in the roots of nontransgenic plants, but its content did not change significantly in transgenic plants (Figure 4B). It is possible that auxin was transported from the shoots to the roots of transgenic plants.

The cultivation of non-transgenic birch plants on a medium with 0.1 mg/L PPT decreased the content of amino acids in leaves, but in transgenic plants, the amount of amino acids increased (Table 1). It is known that the application of PPT leads to a significant depletion of the amino acid pool [44]. In our study, the concentration of PPT was low and, therefore, only a moderate decrease in the amino acid content was observed. However, the amino acid content in transgenic birch plants on a PPT medium was 1.4 times higher compared to nontransgenic plants, which suggests an increase in the resistance to GS inhibitor due to overexpression of the *GS1* gene.

Transgenic plants with the *GS* genes have been repeatedly assessed for resistance to PPT-based herbicides [10,44,45,46], but only a limited resistance to low doses of the herbicide (lower than the field doses) has been shown. Recent studies have shown that the evolution of the resistance to glufosinate in weeds is also associated with *GS* genes—their mutation [47] or overexpression [48]. Some acceleration of the rooting of transgenic birch plants with the *GS1* gene in the presence of PPT also confirms the ability of additional *GS* copies to protect plants from low doses of the herbicide.

### 4.2. Effect of N Availability on Spring Phenology of Birch

The phenology-related traits are of great interest to tree breeders and are particularly relevant in the context of global climate change [49]. It is important to understand how site-specific environmental characteristics other than climate, e.g., soil N, affect tree responses to climate warming [50]. We intended to evaluate the applicability of transgenic birch plants with improved NUE for drought, but first, we set out to assess the effect of N availability on budburst. Plants were grown with a different N availability and, by the end of the third season, transgenic plants significantly exceeded the control in terms of the growth rate under conditions of N deficiency, but not with its abundance (Table 2). A similar effect on plant biomass after N metabolism gene transfer has already been repeatedly noted [51,52,53]. In the spring of the fourth year, we evaluated the budburst and found that the timing of bud break did not depend on the N availability in soil but affected the N-metabolism-modified genotype.

Changes in N availability can influence the physiology of plants, especially in the long term [25]. In autumn, most N from senescing leaves is used for synthesis of bark storage proteins, which serve as a source of N for developing buds and new shoots in spring [7]. Research into the effect of soil N on spring phenology of trees is limited and contradictory. The N supply of *Picea abies* seedlings has not affected the budburst in the greenhouse, but outdoors, the maximum dose of N has accelerated the budburst as compared with lower doses [54]. The effect of the site has also been shown in studies with *B. pendula*. A three-year pot experiment has shown that N fertilization delays the budburst in comparison with the control [55]. However, three years of field growth have shown the opposite result: with increased field availability of N, buds opened faster [50]. The inconsistency of the results can be explained by the influence of the plant species, dose of N, plant age, and growing conditions (greenhouse or field). Our study is consistent with the work of Koenig and colleagues, which did not show any correlation of mean budburst date with soil N availability in *Quercus lobata* [56]. However, buds of transgenic birch plants with the *GS1* gene opened later, which suggested a relationship between N metabolism and phenology. It should be noted that in none of these studies on N availability in soil was the hormone content in the buds evaluated.

### 4.3. Hormonal Status of Birch Buds during Dormancy and Budburst

Phytohormones play an important role in regulating the dormancy–growth cycle, and ABA is one of the most important [57]. Its role in these processes has been shown in a number of fruit and forest trees, including birch [58,59]. Numerous studies have shown that endogenous ABA decreases with dormancy release [21,60]. IAA is also involved in dormancy release in many species. An increase in IAA at dormancy release has been reported for forest [61] and fruit [62,63] trees.

In our study, the content of ABA in transgenic and wild-type birch plants at sufficient N supply decreased after the dormancy release, as previously reported for birch [58,64]; however, it did not change in the control under conditions of N deficiency. Changes in the content of IAA at dormancy release were atypical: it grew only for the control under sufficient N, and it had a small influence, whereas under dormancy, in transgenic plants with N deficiency, it was abnormally high and decreased after the bud break to the level of nontransgenic plants. Birch control plants grown on different N availabilities differed in the contents of both IAA and ABA in the buds, but their budburst rates were similar. On the other hand, transgenic birch plants did not differ in the content of ABA from the control at 10 mM N supply, and in IAA from the control at zero N, but their buds broke later. This suggests that the rate of budburst was associated not only with the ABA and IAA contents but also with other factors, too.

Our experiments with birch plants in vitro revealed a relationship between the increased activity of GS and the accumulation of auxins in shoots, stimulating plant rooting. It can be assumed that the level of auxins in the tissues of transgenic three-year-old birch plants also increased, partly due to the supply of auxins from the buds (the main site of auxin synthesis). This is confirmed by a decrease in the auxin content in buds of GS 8b at dormancy release. As the growth of transgenic birch exceeded the control under conditions of N deficiency, this finding supports the assumption of Lu et al. [14] that enhanced biosynthesis of IAA due to the overexpression of the *PsnGS1.2* gene could play a significant role in enhancing the growth of transgenic tobacco.

The process of bud breaking has been studied mainly in the context of apical dominance, when the removal of the shoot apex reduces the downward flow of auxin and promotes the outgrowth of axillary buds [65,66]. According to the auxin transport canalization model, auxin export from the bud to the stem is a prerequisite for bud outgrowth. However, experiments with the local inhibition of auxin transport from the bud have found that the outgrowth of the bud begins in the absence of auxin efflux from it, and auxin canalization is required a little later for the development of the vascular network to maintain continuous growth and development of the bud [67]. Thus, it is obvious that auxin efflux plays a role in budding. Consequently, a lower level of IAA in the buds in the phase of the appearance of green foliage in GS 8b plants (Figure 6) can indeed be the result of hormone efflux from the buds occurring during their outgrowth.

**Figure 8:**
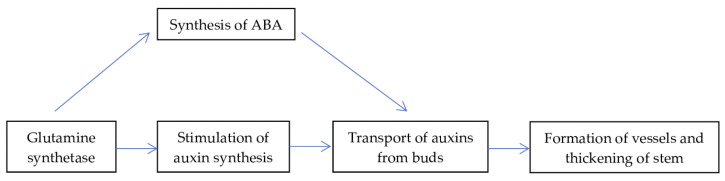
Presumable interaction of GS and ABA in regulation of birch shoot growth.

In nontransformed plants, in the phase of the appearance of green foliage in the absence of N supply, the level of ABA remains high—on the level of dormant buds (Figure 7). At the same time, the concentration of ABA in the buds of transgenic plants is reduced. It is known that GS inhibits the accumulation of ABA [68]. Therefore, a lower level of ABA in transgenic plants (as compared with the control without N supply) in the phase of the appearance of green foliage can be explained by the increased expression of the *GS* gene. Elevated levels of ABA can reduce the process of auxin transport from buds, which are the main sites of auxin synthesis. Auxins are necessary for the normal development of the vascular system of the stem, thereby ensuring its thickening [69]. Thus, we have the following scheme (Figure 8):

Due to this mechanism, ABA can act as an inhibitor of the growth processes of the above-ground part of plants. In our experiments, in the phase of the appearance of green foliage with N deficiency in the buds of nontransformed plants, the concentration of ABA was higher than in the buds of line GS 8b, where ABA was at the level of plants with N supply. An increase in the level of ABA is a well-described reaction of plants to a deficiency of nutrition, including N [70]. This can explain the increased level of this hormone in the buds of the control plants that did not receive N. On the other hand, the absence of ABA accumulation in transgenic plants with N deficiency could contribute to the increased growth of shoots and promote the formation of phenotypic features of transformed plants.

In buds of transgenic birch plants under conditions of N deficiency at dormancy, the content of ABA significantly exceeded the control. As ABA plays a central role in the adaptation of plants to drought [22], an increase in the drought resistance of transgenic birch plants is possible. This has already been reported for plants with the overexpression of *GS* genes [8,10], but no mechanisms have been detailed.

## 5. Conclusions

Our study showed an altered hormonal balance in birch plants overexpressing a conifer cytosolic *GS* gene. During rooting in vitro, transgenic microshoots contained significantly more IAA compared to the control plants. We believe that the reason was the overproduction of GS, since the addition of PPT, a GS inhibitor, reduced the auxin content. Compared to wild-type birch plants, 3-year-old transgenic plants demonstrated enhanced growth and altered ABA content in the buds under conditions of N deficiency, but not its supply. It is likely that the differences in the contents of IAA and ABA in the buds led to a delay in budburst of transgenic birch plants. The results obtained will be used as a basis for future research, in which we plan to study changes in the hormonal profile of various birch genotypes, including transgenic ones, under the influence of drought stress, both separately and in combination with N deficiency. This will enable a better insight into the interaction between phytohormones and nutrients, as well as into the role of genotypic variation in the population of woody plants, in their responses to climate change.

## Figures and Tables

**Table 1:** Contents of amino acids in leaves of the wild-type and transgenic (GS 8b) birch plants (mg/g; amino acids whose contents in transgenic plants significantly increased as compared to the control are bolded).

Amino Acids	WT		GS 8b	
	0 PPT	0.1 PPT	0 PPT	0.1 PPT
Lysine	13.6	4.3	6.1	7.7
Histidine	10.2	8.9	8.1	8.8
Arginine	70.0	46.2	48.4	129.5
**Aspartic acid**	**79.1**	91.3	**102.5**	101.5
Threonine	13.0	13.3	14.8	11.4
Serine	64.0	53.7	50.4	53.6
**Glutamic acid**	**208.0**	180.3	**233.2**	251.4
Glycine	10.2	12.8	8.4	9.5
Alanine	59.6	42.4	48.2	31.2
Valine	8.0	4.7	6.5	10.4
Methionine	3.0	4.1	3.5	3.6
Isoleucine	5.7	4.1	5.5	4.8
Leucine	7.6	5.6	6.6	6.0
Tyrosine	16.0	13.2	18.4	15.9
Phenylalanine	11.5	14.4	14.5	9.5
Asparagine	38.3	24.7	26.0	27.9
**Glutamine**	**70.0**	43.9	**102.6**	84.8
α-Amino butyric acid	17.1	19.2	20.8	68.5
Total amino acids	704.9	587.0	724.5	836.1

**Table 2:** Effect of N supply on the growth of three-year-old birch plants.

Genotype	Height, cm		Stem Base Diameter, mm		Stem Volume, cm^3^	
	N-0	N-10	N-0	N-10	N-0	N-10
WT	140.3 *	185.3 ns	13.7 **	18.1 ns	68.6 *	157.6 ^ns^
GS 8b	161.0	190.7	14.8	17.8	92.9	158.8

Significance of differences measured by *t*-test (*: *p* < 0.05; **: *p* < 0.01; ns: not significant).

## Data Availability

Data are available upon reasonable request.
